# Severe recurrence of reactive infectious mucocutaneous eruption with extensive ocular involvement in an adult due to SARS-CoV-2

**DOI:** 10.1016/j.jdcr.2022.12.026

**Published:** 2023-02-16

**Authors:** David Wu, Ernest Y. Lee, Jacob Lifton, Shannon W. Zullo, Kira Seiger, Rangarajan Nadadur, Lindy P. Fox, Daniel J. Escobar, Allison S. Dobry, Madeline Yung, Kirsten N. Kangelaris, Ryan Y. Arakaki

**Affiliations:** a School of Medicine, University of California San Francisco, San Francisco, California; b Medical Scientist Training Program, University of California San Francisco, San Francisco, California; c Department of Dermatology, University of California San Francisco, San Francisco, California; d Department of Ophthalmology, University of California San Francisco, San Francisco, California; e Department of Medicine, Division of Hospital Medicine, University of California San Francisco, San Francisco, California; f Department of Medicine, Division of Infectious Disease, University of California San Francisco, San Francisco, California

**Keywords:** COVID-19, MIRM, RIME, SARS-CoV-2

## Introduction

Mucocutaneous eruptions are associated with various pathogens, including *Mycoplasma pneumoniae*, herpes simplex virus (HSV), and influenza, most commonly presenting in younger populations. Here we present a case of an adult with recurrent reactive infectious mucocutaneous eruption (RIME) with extensive ocular involvement, triggered by SARS-CoV-2.

## Case report

A 40-year-old man presented to the emergency department with bilateral conjunctival injection and associated purulent discharge, painful oral erosions, vesiculobullous rash, and fever to 103.5 F. Two weeks prior, he antigen-tested positive for COVID-19 and had 4 days of upper respiratory symptoms that self-resolved. Given the severity of presentation and his inability to maintain adequate oral intake, the patient was admitted for further evaluation and management.

This was the fourth and most severe flare of a mucocutaneous eruption dating back 2 years. Each episode had similar findings, including bilateral conjunctival injection 1 week prior to the development of painful lip and mouth erosions. Cutaneous findings were limited in prior episodes, with papules on the distal extremities and no involvement of the genitalia. These prior episodes resolved without a cause identified and did not require treatment with systemic medications.

**Fig 1:**
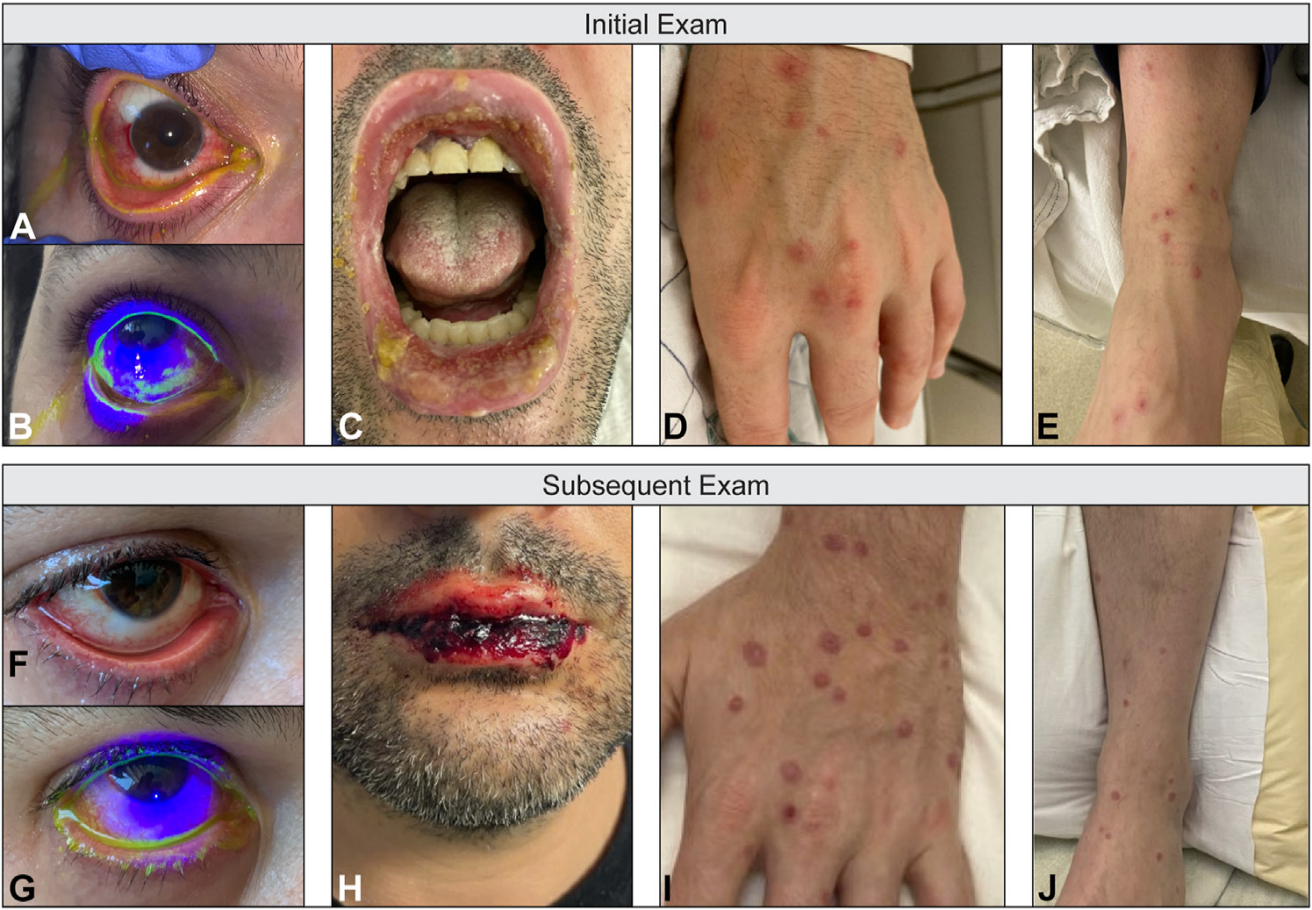
Mucocutaneous findings. Initial exam (**A**-**E**) and subsequent exam on day 4 (**F**-**J**), including ocular fluorescein images. Images show significant bilateral conjunctival injection and large confluent areas of inferior conjunctival ulceration, hemorrhagic crust on the lips, and scattered erythematous papulovesicular lesions with surrounding targetoid erythema on the extremities.

On examination, the patient had scattered erythematous papulovesicular lesions on his trunk and extremities (Fig 1, *A–E*). Lesions were predominantly clustered around the hands and feet and mixed with a few bullae. Erosions and hemorrhagic crusting were noted in the oropharynx, tongue, and lips. No lymphadenopathy was appreciated. Ophthalmic examination revealed scant mucoid discharge, significant bilateral inferior conjunctival injection, and large confluent areas of inferior conjunctival ulceration, but no symblephara formation. The remainder of the patient’s ocular anterior and posterior segment was unremarkable.

Initial laboratory evaluation was notable for leukocytosis and an elevated c-reactive protein. The chest X-ray showed no consolidation. RIME was initially suspected, but erythema multiforme and Stevens–Johnson syndrome/toxic epidermal necrolysis were also considered in the differential diagnosis. The patient was started on intravenous acyclovir, neomycin-polymyxin-dexamethasone ophthalmic ointment every 2 hours, and 1 mg/kg/day of prednisone.

Broad infectious workup produced negative results for blood culture, HSV, varicella-zoster virus, cytomegalovirus, Epstein-Barr virus, Parvovirus B19, chlamydia, gonorrhea, human herpesvirus 6, human herpesvirus 7, HIV, syphilis, leptospirosis, and Hepatitis A/B/C. *M. pneumoniae* polymerase chain reaction (PCR) and IgM testing were both negative, though IgG was positive. SARS-CoV-2 PCR was positive. Skin and conjunctival biopsies were performed and showed vacuolar interface dermatitis. Direct immunofluorescence analysis was negative. Such biopsy findings cannot distinguish between RIME, erythema multiforme, and Stevens–Johnson syndrome/toxic epidermal necrolysis; however, overall clinicopathologic correlation was most consistent with a diagnosis of recurrent RIME triggered by SARS-CoV-2.

The patient’s conjunctival ulceration progressed despite aggressive treatment, extending to the lower palpebral conjunctiva and lid margin of the right eye and involving a large area of the inferior bulbar conjunctiva of the left eye (Fig 1, *F*–*I*). Conjunctival biopsy of the left eye and local amniotic membrane grafting (AMG) to the affected areas in both eyes were performed on day 5. Vision remained at 20/20.

Acyclovir was discontinued after HSV PCR and serologies returned negative. The patient improved on prednisone and supportive care over 1 week. He was discharged home on a prednisone taper. At dermatology follow-up, the patient’s lesions resolved, and he was successfully tapered off prednisone. The conjunctival ulceration resolved with mild residual superior tarsal scarring. The patient developed elevated intraocular pressure bilaterally secondary to steroid use, which resolved with tapering of topical and oral steroids.

## Discussion

Originally called *M pneumoniae*-induced rash and mucositis,^1^ RIME can be triggered by diverse pathogens,^2^ including *Chlamydophila pneumoniae*, enterovirus, *Streptococcus*, influenza, and—recently—SARS-CoV-2.^3^^,^^4^ The vast majority of RIME cases occur in pediatric populations, with upper respiratory prodromes a week prior to mucocutaneous involvement.^1^ Recurrence is reported in a subset of cases (∼8%), typically with decreasing severity.^1^^,^^5^ Although SARS-CoV-2 has been reported to trigger recurrences of RIME in the pediatric population,^2^ the association of SARS-CoV-2 with recurrent episodes in an adult is rare. Here we present a unique case of RIME triggered by SARS-CoV-2 in a 40-year-old man with recurrent episodes of worsening severity and unusually severe ocular involvement.

Ocular manifestations of RIME are present in up to 82% to 97% of cases,^1^ but few reports detail the ocular course, management, and outcomes. Small case series of pediatric patients^6^^,^^7^ found ocular manifestations including conjunctival injection (100%) and ulceration (20%-100%), ulceration of the eyelid margin (40%-87%), pseudo-membrane formation (6.7%-30%), corneal epithelial defects (0%-10%), and superficial punctate keratitis (14.3%). Treatment typically involves topical steroids and antibiotics, with escalation to AMG in refractory cases. Unlike in erythema multiforme or Stevens–Johnson syndrome/toxic epidermal necrolysis, long-term ocular sequelae of RIME are rare and usually mild, but the potential still exists for cicatricial changes leading to blepharitis (13.3%), thickening or scarring of the lid margins (6.7%), or symblephara (6.7%). Of the few reports of RIME triggered by SARS-CoV-2, only 3 mention ocular involvement: all occurred in adolescents who had either mild conjunctivitis or discharge.^4^ Our patient, in contrast, had conjunctival injection and ulceration, as well as progressive sloughing of his lid margin and bulbar conjunctiva requiring AMG, making this the most severe report of ocular RIME induced by SARS-CoV-2. Our patient ultimately responded well to treatment with only mild scarring of the superior tarsal conjunctiva.

Recurrence of RIME can be triggered by distinct pathogens,^2^^,^^5^ which was likely in this case given negative SARS-CoV-2 testing in the past and *M pneumoniae* IgG-positivity in this admission. Estimates of recurrence rates vary,^1^^,^^8^ though severity generally decreases in subsequent episodes.^2^^,^^8^ However, episodes worsened in this case, culminating in a week-long hospitalization. The reason for this severe recurrence is unclear, and likely includes both host and pathogen factors.

The high health care utilization during this recurrence emphasizes the importance of long-term management. Strategies include immunomodulation (eg, mycophenolate mofetil or adalimumab) or rescue therapy during flares (eg, prednisone or etanercept^9^). An unusual and previously undescribed feature present in our case is the specific prodrome marked by bilateral conjunctival injection—as opposed to upper respiratory illness—preceding the generalized mucositis. Given this consistent and early indicator of recurrence, the patient preferred rescue therapy for long-term management. Overall, this case highlights a severe manifestation of recurrent RIME induced by SARS-CoV-2 with multiple unique features.

## Conflicts of interest

None disclosed.
